# Supplementary material for: Supporting the Community’s Health Advocates: Initial Insights into the Implementation of a Dual-Purpose Educational and Supportive Group for Community Health Workers
Source: Healthcare (Basel). 2025 Dec 15;13(24):3288. doi: 10.3390/healthcare13243288 (PMC12732857; doi:10.3390/healthcare13243288)
Supplement: Supplementary file 1 [file healthcare-13-03288-s001.zip › healthcare-3901070-supplementary.pdf]

Supplementary Materials

S1: Post-Session Polls:

1. I feel today's session addressed the following competencies: *(multiple choice)*
   a. Advocacy and community capacity building skills
   b. Effective oral and written communication skills
   c. Cultural competency
   d. Understanding of ethics and confidentiality issues
   e. Knowledge of local resources and system navigation
   f. Care coordination support skills
   g. Teaching skills to promote healthy behavior change
   h. Outreach methods and strategies
   i. Understanding of public health concepts and health literacy
2. Please list one new piece of information that you've learned today. *(long answer)*

S2: 2024 and 2025 Lunch and Learn Participant Survey

Notes:

- *Questions were repeated annually, with the year updated as applicable.*
- *Questions marked [2025 only] were added in the 2025 version to capture additional feedback on program structure and impact.*

1. What are your goals in participating in the CHW Lunch and Learns (drag to rank order with 1 being the most important goals and 5 being the least important goal)?
   i. Problem solving/case work sharing
   ii. Earning continuing education credits
   iii. Support from fellow CHWs
   iv. Networking
   v. Learning about resources
2. What other goals (if any) do you have for participating in the Lunch and Learns? *(short answer)*
3. What areas would you like additional information on during the Lunch and Learn series in 2025 *(drag to rank order with 1 being the most important and 5 being the least important)*?
   i. Professional development
   ii. Health topics
   iii. Community resources
   iv. Support for CHWs
   v. Medical system information
4. Do you have recommendations of topics that you would like covered at the Lunch and Learn in [2024/2025]? *(short answer)*
5. How can we best support you and cover any areas you might need? *(short answer)*
6. [2025 only] We are considering changing the format of the Lunch and Learn but would like your input and feedback before doing so. In order to foster support for the CHW workforce, we are hoping to focus one Lunch and Learn a month on helping problem-solve specific concerns. The structure of our program would change to the following:

On the 2nd Wednesday each month, we will hold a Lunch and Learn (via zoom) during which we have a presenter share on a topic or about a resource. This structure will be the same format as the Lunch and Learns we have been running all year.

On the 4th Wednesday of each month, we will hold a CHW support space. During this time, we will take 30 min to discuss specific concerns CHWs in this group may be facing. Then, we will spend 30 min troubleshooting problems and following up on from previous challenges that were discussed.

Do you support this formatting change

   i. No

ii. Yes

7. [2025 only] For those who participated in the Lunch and Learn series in 2024, what did you like best about the programming? *(short answer*
8. What would you want to change about the Lunch and Learn programming in the upcoming year? *(short answer*
